# A new species of *Amphibulus* Kriechbaumer (Hymenoptera, Ichneumonidae, Cryptinae) from Beijing with a key to species known from the Oriental and Eastern Palaearctic regions

**DOI:** 10.3897/zookeys.312.5294

**Published:** 2013-06-26

**Authors:** Shi-Xiang Zong, Shu-Ping Sun, Mao-Ling Sheng

**Affiliations:** 1 The Key Laboratory for Silviculture and Conservation of Ministry of Education, Beijing Forestry University, Beijing 100083, P. R. China; 2 General Station of Forest Pest Management, State Forestry Administration, 58 Huanghe North Street, Shenyang 110034, P. R. China

**Keywords:** Phygadeuontini, *Amphibulus*, new species, key, taxonomy, China

## Abstract

A new species, *Amphibulus melanarius* Zong, Sun & Sheng, **sp.n.**, belonging to the tribe Phygadeuontini of the subfamily Cryptinae (Hymenoptera: Ichneumonidae), collected from Beijing, China, is reported. A key to the species of the genus *Amphibulus* Kriechbaumer, 1893 known in the Oriental and Eastern Palaearctic Regions is provided.

## Introduction

*Amphibulus* Kriechbaumer, 1893, belonging to the subtribe Endaseina of tribe Phygadeuontini (Hymenoptera, Ichneumonidae, Cryptinae), comprises 26 described species (Yu et al. 2012), of which two are from the Oriental Region (one also from the Eastern Palaearctic Region) (Luhman 1991), three from the Eastern Palaearctic, two from the Western Palaearctic, six from the Nearctic, 15 from the Neotropical and one from the Afrotropical Regions (Yu et al. 2012).

The European species of the genus *Amphibulus* were revised by Sawoniewicz (1990). A revision with 22 new species and a key to the world species was produced by Luhman (1991). Two species have been reported from China (Luhman 1991; Sheng 1999, 2009). A key to the genera of Endaseina found in China, including *Carinityla* Sheng & Sun, 2010, was provided by Sheng and Sun (2013). The status of *Amphibulus* was defined by Townes (1970) and Luhman (1991).

In this article a new species of *Amphibulus*, collected in Beijing, situated at the southern border of the Eastern Palaearctic part of China, is reported.

## Materials and methods

Specimens were collected with intercept traps (Li et al. 2012) in the forests of Mentougou and Yanqing, Beijing (CHINA). The forest of Mentougou is composed of mixed deciduous angiosperms and evergreen conifers, mainly comprising *Pinus tabuliformis* Carr., *Larix gmelinii* var. *principis-rupprechtii* Mayr, *Larix principis-rupprechtii* Mayr, *Betula dahurica* Pall., and *Quercus wutaishanica* Blume. The forest of Yanqing is composed of mixed deciduous angiosperms, mainly *Lespedeza bicolor* Turcz., *Vitex negundo* var. *heterophylla* (Franch.) Rehd., *Spiraea teniana* var. *mairei* (H. Lév.) L. T. Lu, *Ulmus pumila* L., *Populus* spp., *Salix* spp., and a few *Pinus tabulaeformis* Carr. and *Platycladus orientalis* (L.).

Images of whole bodies were taken using a CANON Power Shot A650 IS. Other images were taken using a Cool SNAP 3CCD attached to a Zeiss Discovery V8 Stereomicroscope and captured with QCapture Pro version 5.1. Morphological terminology is mostly based on Gauld (1991). Wing vein nomenclature follows Mason (1986, 1990).

Type specimens are deposited in the Insect Museum, General Station of Forest Pest Management (GSFPM), State Forestry Administration, Shenyang, People’s Republic of China.

## Taxonomy

### Amphibulus

Kriechbaumer, 1893

http://species-id.net/wiki/Amphibulus

Amphibulus Kriechbaumer 1893. Entomologische Nachrichten, 19(8): 122. Type-species: *Amphibulus gracilis* Kriechbaumer.

#### Diagnosis.

*Amphibulus* can be distinguished from all other genera of Endaseina by the combination of the following characters: lower tooth of mandible shorter than upper tooth; median tubercle at upper edge of face small and rounded; posterior edge of mesoscutum with transverse suture, which is unusually conspicuous and complete; scutoscutellar groove without median longitudinal carina; sternaulus reaching to posterior margin of mesopleuron, anterior half deep and often sculptured; median dorsal carina of first tergite weak or absent.

#### Key to species of *Amphibulus* Kriechbaumer known from the Oriental and Eastern palaearctic regions

**Table:** 

1	Male	2
–	Female	5
2	Area spiracularis and area lateralis combined. Tergites 2–6 orange. (Pakistan)	*Amphibulus salicis* Luhman
–	Area spiracularis and area lateralis separated by distinct carina. Tergites 2–6 black, or at most tergites 2–3 orange	3
3	Lower end of occipital carina joining hypostomal carina at base of mandible. Fore wing with ramulus present as stub or swelling. Tergites 2–3 mostly orang. (Female unknown) (Korea)	*Amphibulus bicolor* Luhman
–	Lower end of occipital carina joining hypostomal carina distinctly above base of mandible. Fore wing ramulus absent or at most present as a slight swelling. Tergites 2–3 black or mostly black	4
4	Flagellomeres 10 and 11 with distinct tyloids. Propodeum without apophysis, area superomedia about as long as wide. Apices of tergites 2–6 yellowish. (China, Japan)	*Amphibulus orientalis* Luhman
–	Flagellomeres 10 to 13 with distinct tyloids. Propodeum with apophysis, area superomedia distinctly longer than wide. Apices of tergites 2–6 entirely black. (Female unknown) (China)	*Amphibulus albimaculatus* Sheng
5	Area spiracularis and area lateralis combined. Clypeus about 2.5× as wide as long. Coxae black. Tergites 2–6 orange.	*Amphibulus salicis* Luhman
–	Area spiracularis and area lateralis separated by carina. Clypeus at least 3.0× wider than long. Coxae red, orange or brown, or at least with orange or brown spots. Tergites 2–6 black, dark orange or brown	6
6	First sternite reaching level of spiracle; postpetiole approximately as wide as long. Body mostly brownish or dark orange. Flagellomeres 9–11 white. Median portion of hind tarsus pale yellowish	*Amphibulus orientalis* Luhman
–	First sternite reaching to about half way to spiracle; postpetiole approximately 1.4× as wide as long. Body almost entirely black. Flagellomeres 5–10 (11) white. Hind tarsus brown to brownish black. (Male unknown) (China)	*Amphibulus melanarius* Zong, Sun & Sheng, sp.n.

### Amphibulus melanarius

Zong, Sun & Sheng sp. n.

urn:lsid:zoobank.org:act:2173C040-6C15-45ED-962F-46E47F511D50

http://species-id.net/wiki/Amphibulus_melanarius

Figures 1–11

#### Etymology.

The specific name is derived from the body being entirely black.

#### Types.

Holotype, female, CHINA: Mentougou, 800 to 900 m, Beijing, 22 June 2011, leg. Shi-Xiang Zong. Paratypes: 4 females, same data as holotype except 15 to 22 June 2011. 1 female, CHINA: Songshan Natural Reserve, 672m, Yanqing, Beijing, 27 June 2011, leg. Shi-xiang Zong. 2 females, same data as holotype except 9 June 2012.

#### Diagnosis.

Clypeus 3.0 to 3.3× as wide as long. Area spiracularis and area lateralis separated by carina. First sternite reaching about half distance to spiracle. Postpetiole approximately 0.75× as long as wide, with dense longitudinal wrinkles. Head except mandibles and clypeus, mesosoma and all tergites black. Flagellomeres 5 to 10 (11) white. Coxae red.

#### Description.

Female (Figs 1–11). Body length 6.5 to 7.5 mm. Fore wing length 5.5 to 6.0 mm. Ovipositor sheath length approximately 1.0 mm.

**Head.** Face and clypeus with dense brown hairs. Face (Fig. 2) 2.6 to 2.7 times as wide as long, median portion slightly convex; with dense punctures. Clypeus 3.0 to 3.3× as wide as long, basal half with dense punctures, apical half smooth and shiny, with unclear, indistinct striations; subapical portion distinctly raised as a transverse ridge; apical margin deeply depressed, median section almost truncate. Mandible (Fig. 3) distinctly elongate, shiny, with sparse, fine punctures; base distinctly concave centrally; upper tooth evidently longer than lower tooth. Subocular sulcus indistinct. Malar space 0.55 to 0.6 times as long as basal width of mandible. Gena in dorsal view 1.1 to 1.2× as long as width of eye, with uneven, fine punctures, distance between punctures 0.5 to 4.0× their diameter. Vertex (Fig. 4) slightly shining, with irregular elongate punctures. Postocellar line 0.83 to 0.88× as long as ocular-ocellar line. Upper portion of frons almost flat, with dense punctures, distance between punctures 0.2 to 1.0× their diameter; mediocentral portion with fine transverse wrinkles; lower portion concave, smooth, shiny, with median longitudinal carina. Antenna with 25 or 26 flagellomeres, median portion of flagellum, flagellomeres 5 to 21, slightly widened; apical half cylindric. Ratios of lengths from first to fifth flagellomeres: 1.1:1.0:1.1:0.9:0.8. Apical truncation of scape 15 to 18 degrees from transverse. Occipital carina complete, lower end joining hypostomal carina at base of mandible.

**Mesosoma.** Anterior margin of pronotum with fine longitudinal wrinkles; lateromedian portion with transverse wrinkles; upper posterior portion with punctures, distance between punctures 0.5 to 1.5× their diameter. Anterior and lateral portions of mesoscutum (Fig. 5) smooth, shiny, with fine, sparse, indistinct punctures; posterior median portion with punctures and longitudinal wrinkles. Scutoscutellar groove with fine, short longitudinal wrinkles, without median longitudinal carina. Scutellum (Fig. 7) almost flat, shiny, with sparse, uneven punctures. Postscutellum transversely convex, anterior portion transversely concave; with sparse punctures. Upper and anterior portions of mesopleuron (Fig. 6) with distinct punctures; mediocentral portion rough, with elongate punctures; lower posterior portion with transverse wrinkles. Epicnemial carina reaching to subtegular ridge. Speculum small, smooth, shiny. Metapleuron convex, with dense, elongate punctures. Submetapleural carina complete, anterior end strongly convex. Hind leg (Fig. 8) with dense brown setae. Apical truncation of hind tibia approximately transverse, internal side with dense, fine fringe of setae at the apex (Fig. 8a). Ratios of lengths of first to fifth hind tarsomeres are 3.8:2.0:1.7:1.0:1.5. Fore wing with vein 1cu-a distal to 1/M by about 0.5 to 1.5× width of vein. Areolet (Fig. 9) receiving vein 2m-cu approximately at posterior 0.3. 2m-cu almost vertical, with a wide bulla. Hind wing vein 1-cu 2.3 to 3.0× as long as cu-a. Propodeum (Fig. 7) completely areolated. Area basalis and area superomedia combined, with irregular punctures and short wrinkles; area externa with fine, indistinct punctures; area dentipara with indistinct punctures; area petiolaris with dense elongate punctures; remainder roughly sculptured. Propodeal spiracle small, circular.

**Metasoma** (Fig. 10). First tergite approximately 1.35 to 1.5× as long as apical width. Postpetiole approximately 0.75 as long as wide, with dense, fine longitudinal wrinkles. Dorsolateral and ventrolateral carinae complete. Spiracle circular, small, located approximately at posterior 0.3 of first tergite. Second tergite 0.5 to 0.6× as long as wide, with fine punctures, punctures on posterior portion much sparser and finer than that on anterior portion. Third tergite 0.6 to 0.7× as long as wide, anterior portion with fine, indistinct punctures, posterior almost smooth. Tergites 4 and 5 with dense brown hairs. Ovipositor (Fig. 11) with weak subapical nodus.

**Figures 1–11.**
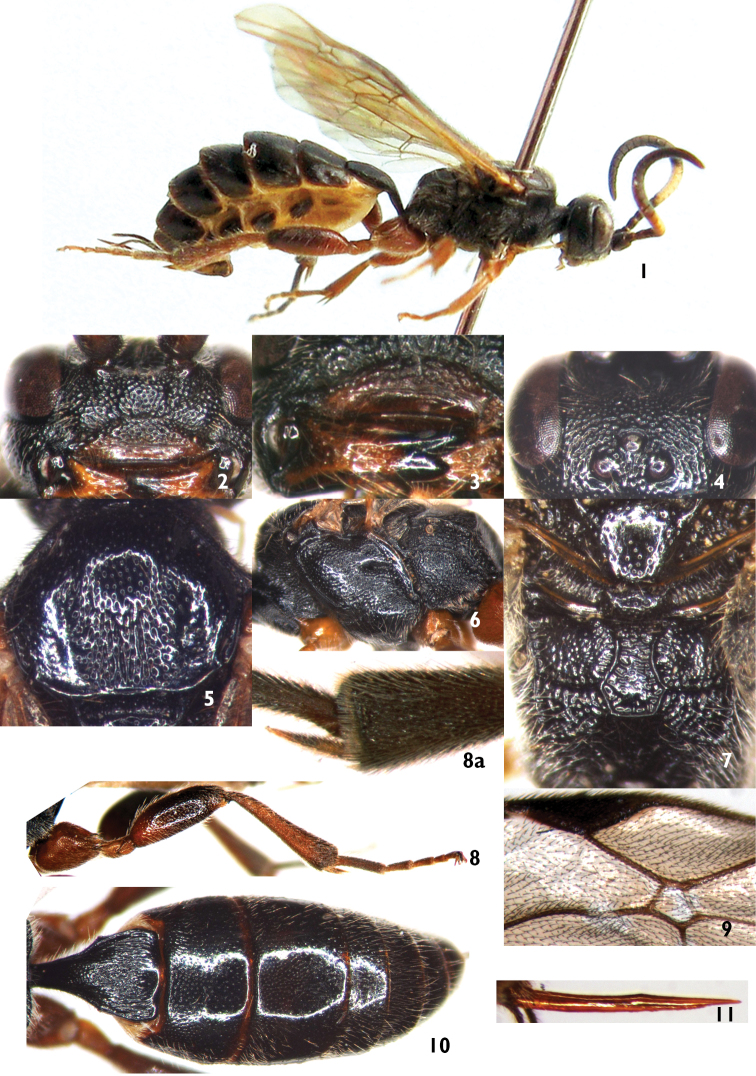
*Amphibulus melanarius* Zong, Sun & Sheng, sp.n. Holotype. Female **1** Habitus, lateral view **2** Head, anterior view **3** Mandible **4** Head, dorsal view **5** Mesoscutum **6** Mesopleuron **7** Scutellum, postscutellum and propodeum **8** Hind leg **8a** Inner apical portion of hind tibia **9** Areolet **10** Metasoma, dorsal view **11** Ovipositor, lateral view.

**Color** (Fig. 1). Black, except the following: anterior profiles of scape and pedicel, basal portion and anterior profile of apical half of flagellum slightly reddish black. Flagellomeres 5 to 10 (11) white. Apical portion of clypeus, mandible except base and teeth, maxillary and labial palpi, tegula, front and middle legs, hind coxa, trochanter, and base and ventral profile of femur, basal portion of hind tibia red to reddish brown. Dorsal surface of hind femur and apical portion of hind tibia dark brown. Hind tarsus brown to brownish black. Pterostigma and wing veins brownish black.

## Supplementary Material

XML Treatment for Amphibulus

XML Treatment for Amphibulus melanarius
